# Architects of nature: growing buildings with bacterial biofilms

**DOI:** 10.1111/1751-7915.12833

**Published:** 2017-08-16

**Authors:** Martyn Dade‐Robertson, Alona Keren‐Paz, Meng Zhang, Ilana Kolodkin‐Gal

**Affiliations:** ^1^ Faculty of Humanities and Social Sciences School of Architecture, Planning and Landscape Newcastle University Newcastle upon Tyne UK; ^2^ Department of Molecular Genetics Weizmann Institute of Science Rehovot Israel; ^3^ Department of Applied Sciences Faculty of Health and Life Sciences Northumbria University Newcastle upon Tyne UK

## Abstract

In his text ‘*On Architecture’*, Vitruvius suggested that architecture is an imitation of nature. Here we discuss what happens when we begin using nature in architecture. We describe recent developments in the study of biofilm structure, and propose combining modern architecture and synthetic microbiology to develop sustainable construction approaches. Recently, Kolodkin‐Gal laboratory and others revealed a role for precipitation of calcium carbonate in the maturation and assembly of bacterial communities with complex structures. Importantly, they demonstrated that different secreted organic materials shape the calcium carbonate crystals formed by the bacterial cells. This provides a proof‐of‐concept for a potential use of bacteria in designing rigid construction materials and altering crystal morphology and function. In this study, we discuss how these recent discoveries may change the current strategies of architecture and construction. We believe that biofilm communities enhanced by synthetic circuits may be used to construct buildings and to sequester carbon dioxide in the process.

## Introduction

Building construction and maintenance constitute a major source of greenhouse gasses. In addition to the upfront environmental costs of construction, buildings tend not to be adaptable to change – requiring for example mechanical heating and ventilation and being subject to costly and materially inefficient dismantling at the end of their lives. Inefficient use of energy and materials in construction poses a major challenge for the next generations and was included in the pivotal resolution adopted by the General Assembly in 2015 – ‘Transforming our world: 2030 Agenda for Sustainable Development’ as Goal 11: Sustainable Cities and Communities. Therefore, one of the main goals of architectural science is the development of alternative, energy‐efficient materials and methods of construction, adaptable to environmental conditions.

Examples of this emerging research include the development of Material Based Design Computation (hereafter MBDC). The term MBDC was defined as ‘*the process of computationally enabled form‐finding, informed by material properties*’ (Oxman and Rosenberg, 2007). The MBDC process makes use of computational modelling of materials with simulations of, for example, loading (e.g. finite element analysis) being used to generate a form for manufacture. Using 3D printers capable of printing in more than one material and varying the density of each material gives the designer tight control over the structure – using materials only where they are necessary. Oxman illustrates this with a chair, which surface is designed so that its variegated stiffness accommodates different levels of ergonomic support. This construction technique allows for the creation of functionally graded materials (Oxman *et al*., 2011). This area of research is often inspired by biological processes, such as bone remodelling in response to forces in the environment. Unlike biological systems, however, computation and construction are yet to be combined. Biological systems are adaptable and evolved to respond to changeable environmental conditions – while computation is done *in silico*. Therefore, the key challenge in building science (and other types of manufacturing research) is to mimic this aspect of biological systems, and perhaps even directly integrate those systems in design and manufacture. This approach may enable to embed computational design *in vivo* in the material itself.

## Biofilms

One biological system that might be of key importance in future development of architecture is the bacterial biofilm. The term biofilm refers to complex heterogeneous structures comprising different populations of microorganisms that attach and form a community on an inert (e.g. rocks, glass, plastic) or organic (e.g. skin, cuticle, mucosa) surfaces (Kolter and Greenberg, 2006). The properties of the surface, such as charge, hydrophobicity and roughness, determine initial bacterial attachment (Palmer *et al*., 2007). A common principle of all biofilms is the production of extracellular matrix (ECM) composed of different organic substances, such as extracellular proteins, exopolysaccharides and nucleic acids (Branda *et al*., 2005). While the ability to generate ECM appears to be a common feature of multicellular bacterial communities, the means by which these matrices are constructed and function are diverse (Branda *et al*., 2005; Steinberg and Kolodkin‐Gal, 2015; Dragos and Kovacs, 2017).

Biofilms offer bacteria several ecological and physiological advantages. They constitute a physical barrier against host defences during infection and protect bacteria from antimicrobial agents (such as disinfectants and antibiotics) by reducing diffusion of those toxic compounds (Landry *et al*., 2006). The biofilm structure and the composition of the ECM (that consists of up to 97% water) protect cells from desiccation (Branda *et al*., 2005; Steinberg and Kolodkin‐Gal, 2015). The ECM mediates surface adhesion, cell to cell communication and self‐organization within the biofilm (Kolodkin‐Gal *et al*., 2013; Zhao *et al*., 2013), as well as structural integrity (Chu *et al*., 2006) and nutrient acquisition (Dragos and Kovacs, 2017).

Biofilm components can serve as important tools in biotechnology. Some ECM compounds may be used in cosmetics, food and pharmaceutical industries (Stanley‐Wall and MacPhee, 2015; Berlanga and Guerrero, 2016). In addition to being a source of proteins with novel functions, biofilms can be produce chemicals by fermentation (ethanol, butanol, lactic acid, and succinic acid), in wastewater treatment and in bioremediation. In industrial applications, such as biofilm reactors, microbial cells are fixed on different supports by entrapment, covalent bond formation or adsorption. The later approach takes advantage of the natural ability of bacterial cells to adhere to a support (such as charcoal, resin, vermiculite, sand particles and polypropylene; Berlanga and Guerrero, 2016).

In this study, we will discuss another unique feature of bacterial biofilms – template driven biomineralization; and its potential application in sustainable architecture and bioconstruction.

## Bacteria‐associated precipitation of calcium carbonate

O*rganic* extracellular matrix production has been extensively studied as a means of cell–cell and cell–substrate adhesion (Branda *et al*., 2001, 2005; Kolter and Greenberg, 2006). Recently, we and others determined that precipitation of calcium carbonate contributes to the assembly of complex biofilm architecture (Li *et al*., 2016a,2016b,c; Oppenheimer‐Shaanan *et al*., 2016).

Bacterial biomineralization is a well‐established phenomenon. For example, *Sporosarcina pasteurii* and *Bacillus megaterium* were shown to promote calcium carbonate precipitation (Dupraz, 2009). While calcium is available from the environment, bicarbonate is actively produced by CO_2_ hydration (CO_2_ + H_2_O ↔ HCO_3_ + H^+^), where the source of CO_2_ can be a byproduct of bacterial metabolism or of the immediate environment (Dupraz *et al*., 2009; Perito and Mastromei, 2011; Dhami *et al*., 2013). The capacity of bacterial mats to sequester CO_2_ actively during biomineralization is of special importance. The concentration of CO_2_ in the atmosphere is constantly rising, with the global annual mean increasing markedly from 280 to 400 ppm as of 2015 (Jain *et al*., 2016). While not toxic, CO_2_ contributes to the greenhouse effect, and therefore to global warming – inducing unprecedented environmental changes (Memmott *et al*., 2007; Socolow and Lam, 2007; Lidbury *et al*., 2012). A continuous increase in near‐surface atmospheric temperature is often reported, and the additional energy stored in the climate system contributes to ocean warming. Taking urgent steps to combat climate change is one of the goals of the Agenda for Sustainable Development, highlighted in Goal 13: Climate Action.

Bacterially induced calcium carbonate precipitation has been proposed as an environmentally friendly method to protect decayed ornamental stone. The method relies on the bacterially induced formation of a calcium carbonate precipitate on limestone. The carbonate cement promoted by bacteria appears to be highly coherent (Vahabi *et al*., 2015), and this technique has been explored for the improvement of the durability of cementitious materials (Park *et al*., 2012). Calcium carbonate bio‐deposition technologies have been used for consolidation of sand columns (Nemati and Voordouw, 2003) and for repair and remediation of cracks in concrete (Pinar *et al*., 2010; Wang *et al*., 2017).

Importantly, our recent observations suggest that the shape and the growth of calcium carbonate crystals within bacterial communities can be predicted and manipulated (Oppenheimer‐Shaanan *et al*., 2016), a function critical for novel applications in the construction industry.

## Controlling the shape of the crystalline calcium carbonate using ECM templates

Growth of calcite crystals occurs in layers (Wang *et al*., 1997). The relative growth rates in the various axes may alter crystal shape and morphology and may be influenced by the biogenic (organic) environment (Weiner and Addadi, 1991), and by organic polymeric substances (McConn and Nakata, 2002). For many biofilm producing bacteria, the identity of the exopolymeric substances and the genes that encode them are resolved. It is known that ECM absorbs Ca^2+^ and promotes calcium carbonate formation by providing nucleation sites (Dupraz *et al*., 2009; Obst *et al*., 2009). However, the exact extra polymeric substances critical for biomineralization and crystal structure remain to be determined. Recently, following our finding that a defect in biomineralization leads to a flaw in colony morphology in *Bacillus subtilis*, we tested whether mineral absorption and assembly are related to the ECM components secreted during biofilm formation (Branda *et al*., 2005). The genetically manipulable *Bacillus subtilis* forms a well‐defined matrix during biofilm assembly (Mielich‐Suss and Lopez, 2015), and therefore, the effect of distinct ECM components on the growing mineral can be studied. The effect of the matrix templates was evaluated by comparing the morphology of calcium carbonate crystals at the following mutants: the *tasA* mutant, impaired in production of secreted amyloids (Romero and Kolter, 2014; Bucher *et al*., 2015), the *eps* and *ywqC‐F* mutants, impaired in different exopolysaccharides synthesis pathways (Mijakovic *et al*., 2003; Branda *et al*., 2004) and a double *eps* and *ywqC‐F* mutant. Calcium carbonate assembly and localization within the wrinkles was significantly compromised in all matrix mutants, consistent with the concept of mineral growth aided by nucleation sites provided by the matrix. None of the mutations had any effect on planktonic growth (Oppenheimer‐Shaanan *et al*., 2016). The analysis of crystal morphology in the different matrix mutants showed that interactions between the macromolecules and the mineral phase can affect the growth of calcium carbonate crystals. Environment Scanning Electron Microscopy (ESEM) revealed that, *in all backgrounds,* crystals contained rod‐shaped pores consistent with the size of the bacteria. Examination of the crystals generated by the wild‐type strain revealed both rough crystal faces and smooth flat crystal faces, demonstrating the direct consequence of the spontaneous atomic organization. The crystals formed in the wild‐type strain displayed elongated prismatic morphology instead of the rhombohedral morphology, the most common form of calcite (de Leeuw and Parker, 1998). Crystals formed by mutants for *tasA* amyloids and *eps* exopolysaccharides were significantly longer. Interestingly, the *ywqC‐F* mutant, defective in secretion of acidic exopolysaccharides, produced calcite of strikingly clear rhombohedral morphology (Gilbert *et al*., 2011), as it had only one plane of symmetry through four vertices, and six smooth rhombic faces. Therefore, in the absence of this exopolysaccharides template, the interference of the organic matter with the crystal growth was minimal. Thus, we demonstrated that the secreted organic matter interferes with crystal growth. The crystals were growing in the C axis, and the organic matter mainly interfered with growth of the crystals towards this axis. The levels of interference were evaluated according to the elongation pattern of the crystal, that is, if the extensions are thinner, then the inhibition is higher.

**Figure 1:**
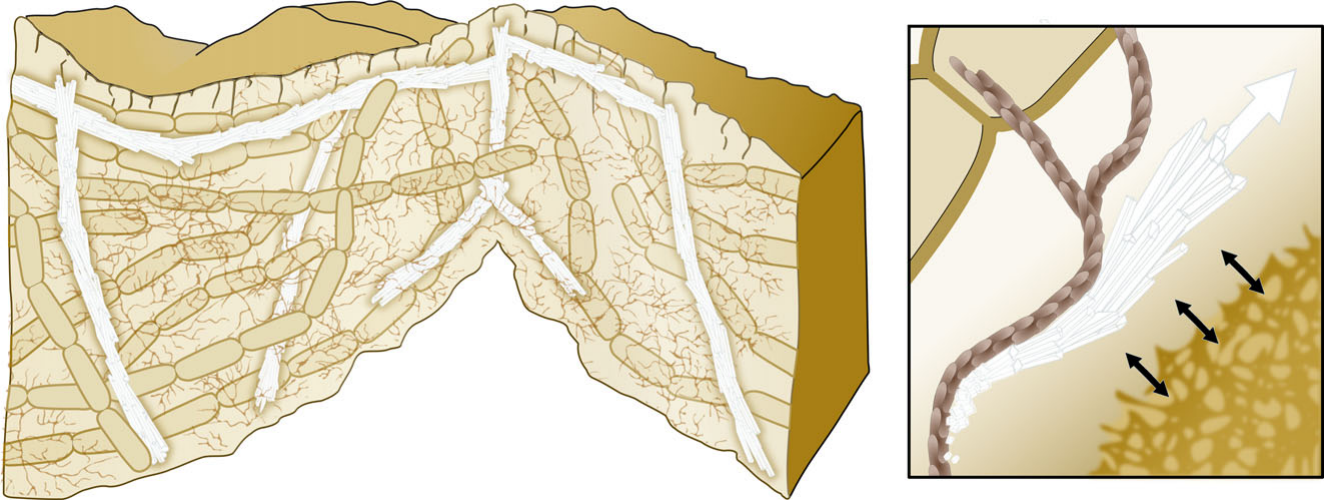
Model for biomineralization‐mediated scaffolding of bacterial biofilms. A directed growth of the calcium carbonate crystals allows mechanical support of the 3D structure. The bacterial extracellular matrix (brown) promotes the crystals' growth in specific directions. This figure was modified from Figure S14 in (Oppenheimer‐Shaanan *et al*., 2016).

The interaction between ECM components and mineral crystals was further assessed and confirmed by Fourier transform infrared (FTIR) analysis of the crystals (Oppenheimer‐Shaanan *et al*., 2016). These results strongly indicate that using artificial overexpression constructs for exopolysaccharides or amyloids, as well as mutant strains placed at critical locations, is likely to generate differential calcium carbonate‐based materials (For an illustration see Fig. 1). Furthermore, they provide a proof of concept for controlling the end product of bacterial construction by providing appropriate ECM mutants for each structural element.

The effect of ECM‐based nucleation on crystal assembly is probably a general feature of biofilm mats. Importantly, the shape of the calcium carbonate crystals varies between different species, such as *Mycobacterium smegmatis* that produces fatty‐acids based ECM (Purdy *et al*., 2013; Oppenheimer‐Shaanan *et al*., 2016), and *Pseudomonas* species containing various polysaccharides in their matrix (Harmsen *et al*., 2010). All these soil bacteria are genetically manipulable and provide a flexible tool‐box for future engineers and architects. In theory, the desired calcium carbonate element to be used in bio‐cement or building foundations can be produced by designing the composition of the bacterial communities.

## Improving construction by coupling calcium carbonate production with shear stress

The role of the foundation of a building is to bear and transfer the building load. The ultimate load which a foundation can support may be calculated using bearing weight capacity. For preliminary design, presumed bearing values can be used to indicate the pressures which would normally result in an adequate factor of safety. For example, the allowable bearing capacity (q_a_) is the maximum bearing stress that can be applied to the foundation, so it is safe against instability due to shear failure and the maximum tolerable settlement is not exceeded. The allowable bearing capacity is normally calculated from the ultimate bearing capacity using a factor of safety (F_s_).

Our vision is to construct bacterial‐based foundations that respond to the changing load of the structure by producing calcium carbonate to cement soils and to control the morphology of crystals so the materials are functionally graded.

Biomineralization is already implicated in large‐scale geotechnical processes. A build‐up of biofilms can have a substantial impact on the geotechnical characteristics of soils – leading to greater adhesion of sediment particles and strengthening the soils against load. By harnessing these processes, it is possible to begin to develop materials relevant for large‐scale building and construction. Bacterially induced calcium carbonate precipitation has already been used to produce ‘self‐healing’ concrete. *Bacillus megaterium* spores and suitable dried nutrients are mixed and applied to steel‐reinforced concrete. When the concrete cracks, water ingress dissolves the nutrients and the bacteria germinate triggering calcium carbonate precipitation, resealing the crack and protecting the steel reinforcement from corrosion (Jonkers, 2007). This process can also be used to manufacture new hard materials, such as bio‐cement (Dosier, 2011).

Currently, the full potential of bacteria‐driven biomineralization is far from being realized, as it is used as a passive filling rather than as a smart designable material. The ultimate goal is to develop ways to control the timing and the location of mineral formation, as well as the physical properties of the mineral itself, by environmental input.

Interestingly, *Bacillus subtilis* has already been shown to respond to its environment, by changing the production of the ECM. It uses the polymers produced by single cells during biofilm formation as a physical cue to coordinate ECM production by the bacterial community (Rubinstein *et al*., 2012; Chan *et al*., 2014). Conceivably, the physical properties of matrix polymers are also exploited for synchronizing development of biofilms.

Determining the bacterial response to physical stress is crucial for predicting the behaviour of the future biofilm foundations. Probing physical properties ordinarily requires applying physical forces: to measure the strength of a material, it needs to be stretched or squeezed. In the case of biofilms, rheological assays that involve measuring either local microscopic strength of *Bacillus subtilis* biofilms formed around the force probe of the rheometer, or probing the macroscopic robustness of the developing pellicle over time under relevant conditions (Rubinstein *et al*., 2012) can be used. This approach is different and complementary to previously established assays in which large biofilms are first grown and then transported to a rheometer for mechanical testing (Jones *et al*., 2011), or alternatively are grown within a confined microenvironment.

Additionally, using polymers as our osmotic agents or mechanical modulators of the environment rather than small molecules that are more commonly used (such as salts and sugars), we can now identify alternative physiological effects on gene expression well as distinguish among various possible physical properties such as viscosity, rigidity and ionic effects (Rubinstein *et al*., 2012).

The current challenge is identifying the relevant mechanical receptors for load stress, and coupling the production of calcium carbonate minerals to them. Encouragingly, bacterial mechanoreceptors that regulate gene expression, such as mechanosensitive channels serving to transport osmoticants (Martinac, 2001) and the osmolality‐sensing protein EnvZ (Wang *et al*., 2012), are consistently exposed. Three independent enzymatic pathways can be placed under mechanical receptors and increase bacterial biomineralization:

- *Urease*. Biomineralization occurs preferentially in alkaline pHs. Alkaline environments promote generation of carbonate ions and negatively charge the functional groups on the bacterial surface (Phoenix and Konhauser, 2008; Dhami *et al*., 2013). Various bacteria produce urea by the degradation of arginine and purines (Vogels and Van der Drift, 1976). Hydrolysis of urea by urease is the most easily controlled mechanism of microbial calcium carbonate precipitation, with potential to produce high quantities of carbonates within a short period of time: 1 mol of urea is intracellularly hydrolyzed to eventually form bicarbonate, 1 mol of ammonium and hydroxide ions, which lead to increased pH. Thus, coupling urease expression to load stress is expected to promote biomineralization locally at the stress source.
- *Carbonic anhydrases*. The enzymes that catalyze the interconversions of carbon dioxide and water to bicarbonate and protons facilitate the formation of calcium carbonate (Lotlikar *et al*., 2013; Muller *et al*., 2013). This family of genes is wide spread in bacterial genomes and can be put under mechanosensor control.
- *ECM genes*. As described above, different matrix templates can provide nucleation sites and promote crystal's growth. Interestingly, many ECM genes are already under the regulation of physical and mechanical cues (Steinberg and Kolodkin‐Gal, 2015).

## Summary

In this study, we explore the potential use of bacteria‐induced biomineralization in architecture and construction. Research of biomineralization promoted by bacterial biofilms is of crucial importance, both as purely scientific study of microbiology and as it promises biotechnology significantly benefits in soil bio‐mediation. Microbiologically induced calcite precipitation can be used to enhance the shear strength of soil for environmental remediation applications. Furthermore, applying beneficial bacteria to the foundations of eco‐friendly buildings and bio‐cement will result in carbon dioxide sequestration in the form of a functional mineral. Thus, incorporating bacterial biofilms that form calcium carbonate into our construction materials provides a sustainable solution for building future cities, as well as for the critical issue of global warming – taking us one step closer to achieving the Sustainable Development Goals as defined in the 2030 Agenda for Sustainable Development.

The work described here represents an emerging interdisciplinary field combining applied microbiology with building science research. Several pioneering projects have already proved the potential utility of microorganisms in the production and maintenance of building materials. The ultimate goal is to design new ‘intelligent’, adaptable, energy‐efficient materials, by integrating living cells into building and architecture.

The roman architect Marcus Vitruvius Pollio suggested that architecture is an imitation of nature. Biofilms formed by soil bacteria evolved to deal with the challenges of carbon dioxide emissions from cellular respiration, and to self‐renew following load stresses. Now it is time that the natural solutions bacteria found are included in the architectural ‘tool box’ of the future.

## Conflict of Interest

None declared.
